# Current Bioequivalence Study Designs in South Korea: A Comprehensive Analysis of Bioequivalence Study Reports Between 2013 and 2019

**DOI:** 10.3389/fphar.2021.651790

**Published:** 2021-05-04

**Authors:** Ki Young Huh, Eunwoo Kim, Soyoung Lee, Hyounggyoon Yoo, Seonghae Yoon, Kyung-Sang Yu, Jae-Yong Chung

**Affiliations:** ^1^ Department of Clinical Pharmacology and Therapeutics, Seoul National University College of Medicine and Hospital, Seoul, South Korea; ^2^ Department of Clinical Pharmacology and Therapeutics, CHA University Bundang Medical Center, CHA University School of Medicine, Gyeonggi-do, South Korea; ^3^ Department of Clinical Pharmacology and Therapeutics, Seoul National University College of Medicine and Bundang Hospital, Gyeonggi-do, South Korea

**Keywords:** bioequivalence, regulations, generic drug, Intrasubject coefficient of variation, biopharmaceutics classification system

## Abstract

Demonstration of bioequivalence (BE) is mandatory while developing generic drugs. The scientific concept of BE applies equally to different regulatory agencies. However, the application of the concept may differ for each agency, which can affect the design of BE studies. To evaluate the study practices in terms of the BE concept in South Korea, we retrospectively analyzed BE study reports available from Ministry of Food and Drug Safety between 2013 and 2019. Statistical estimation of the pharmacokinetic parameters, including peak concentration and area under the concentration–time curve to the last measurable concentration, as well as study design, number of subjects in a study, study duration, fasting status, and formulation of specific drugs were obtained. The drugs were classified per World Health Organization Anatomical Therapeutic Chemical Classification and Biopharmaceutics Classification System. Post-hoc intrasubject coefficient of variation and corresponding sample sizes were calculated from the 90% confidence intervals of pharmacokinetic parameters. A total of 143 generic drugs in 588 BE studies were analyzed. The largest number of studies were performed in the area of Cardiovascular system (172 studies), followed by Nervous system (143 studies) and Alimentary tract and metabolism (92 studies). Overall, BE studies in South Korea were conducted in accordance with the global guideline despite the differences in details. BE studies were focused on the several therapeutic areas and conducted in a similar manner. The number of subjects was generally larger than that estimated with 90% power.

## Introduction

Development of generic drugs is one of the effective strategies to increase patient access to therapeutic drugs. Regulatory agencies have adopted an abbreviated approval process for generic drugs (Sravika et al., 2017). Demonstration of bioequivalence (BE) is required for approval of generic drugs, instead of repeating clinical trials on safety and efficacy (Lee et al., 2016). As a result of this abbreviated approval process, generic drugs can be supplied at lower cost. For instance, generic drug accounted for only 27% of total prescription costs in the United States despite a large proportion (89%) in the total prescription cost (Howard et al., 2018).

The scientific concept of BE is defined uniformly across various regulatory agencies (Chen et al., 2018). BE is achieved when the bioavailabilities of two drugs “lie within acceptable predefined limits” to ensure “similarity in terms of safety and efficacy” (European Medicines Agency, 2010), thus demonstrating “the absence of significant difference in the rate and extent of absorption under similar experimental conditions” (U.S. Food and Drug Administration, 2013; Cristofoletti et al., 2018). BE can be demonstrated *in vivo* and *in vitro* (Chow, 2014), although *in vitro* assessment has limited acceptance, i.e., only for drugs with high solubility and permeability (Cristofoletti et al., 2013). A standard approach for demonstrating BE is a two-way crossover (2 × 2) clinical trial conducted in healthy subjects (Chow, 2014).

Although the concept of BE is accepted globally, regulatory requirements and standards for BE are not consistent among countries (Davit et al., 2013; Kaushal et al., 2016; Chen et al., 2018). The difference is observed even among the members of International Council for Harmonization of Technical Requirements for Pharmaceuticals for Human Use (ICH) (Davit et al., 2013; Kuribayashi et al., 2016). The difference lies in terms of recommended study design, method for pharmacokinetic (PK) parameter estimation, and modification of BE criteria for highly variable drugs (Davit et al., 2013).

As one of the ICH members, South Korea follows global standards for BE (Davit et al., 2013). BE studies in South Korea are regulated by Ministry of Food and Drug Safety under Standard on Pharmaceutical Equivalence Test (Ministry of Food and Drug Safety, 2014). Currently, the Ministry of Food and Drug Safety only accepts domestic BE study results (Ministry of Food and Drug Safety, 2018b). Furthermore, generic products approved in other countries are required to submit BE study results from the authorized study centers in South Korea for approval (Ministry of Food and Drug Safety, 2018b).

The standard for BE studies in South Korea has been comprehensively reformed since 2006 to be at par with the global standards (Ryu and Kim, 2017). However, specific regulatory requirements are either absent or different from those of other countries. Several important criteria for evaluation of BE in South Korea are as follow (Ministry of Food and Drug Safety, 2018a):

• When blood samples are used, the comparative evaluation parameters include AUC_t_ and C_max_ in a single dose study, and AUC_τ_ and C_ss,max_ in a multiple-dose study. [Article 17 (Lee et al., 2016)]
• When log transformation and statistical evaluation on comparative parameters of the reference and test product except T_max_ are performed, the 90% confidence intervals for the difference in mean values between the test and reference should be within log 0.8 to log 1.25. [Article 17 (Howard et al., 2018)]
• Blood collection shall be conducted with sufficient time period of more than 3 times the elimination half-life or AUC_0-t_ to reach at least 80% of AUC_∞_. [Article 15 (Lee et al., 2016)]
• The number of subjects shall be based on an appropriate sample size calculation and may be added or subtracted depending on types and characteristics of active ingredients. The number, in principle, shall be at least 12 or more. (Article 13)

(Abbreviations: AUC_∞_, area under the drug concentration in blood-time curve from zero to infinity; AUC_t_, area under the drug concentration in blood-time curve from zero to the final sampling time t; AUC_τ_, area under the drug concentration in blood-time curve over one dose interval at steady-state; C_max_, the maximum drug concentration in blood; C_ss,max_, The maximum drug concentration in blood at steady state; T_max_, Time to the maximum drug concentration in blood.)

In the light of the global concept of BE, we evaluated the BE studies conducted in South Korea by retrospectively analyzing the BE study reports available from Ministry of Food and Drug Safety between 2013 and 2019.

## Materials and Methods

### Data Collection

BE study reports from March 2013 to November 2019 were collected from the public database of Ministry of Food and Drug Safety and analyzed retrospectively. In South Korea, the BE study reports are provided only for generic drugs that have demonstrated BE. All studies were conducted in accordance with Standard on Pharmaceutical Equivalence Study and were approved by Ministry of Food and Drug Safety.

Following statistics for PK parameters were obtained from the BE study reports for test and reference products:

• Maximum plasma concentration (C_max_): mean and standard deviation, test-to-reference geometric mean ratio (GMR), and 90% confidence interval (CI)
• Area under the concentration–time curve from zero to the last measurable point (AUC_last_): mean and standard deviation, test-to-reference GMR, and 90% CI
• Elimination half-life (t_1/2_): mean and standard deviation
• Time to reach C_max_ (T_max_): median, minimum, and maximum

The study information obtained from the reports included design (e.g., 2 × 2, 2 × 2 × 4), fasting status, study duration (duration up to the last PK sampling point), and the number of subjects analyzed. Brand names for test and reference drugs were obtained and standardized to generic names. Additionally, information was collected regarding the dose strength, fixed-dose combination, modified-release dosage form [e.g., extended-release (ER), controlled-release (CR) form], and route of administration other than oral administration (e.g., patch).

Each generic drug corresponded to one BE study. Each drug was classified per World Health Organization Anatomical Therapeutic Chemical Classification system. Active pharmaceutical ingredients for fixed-dose combination products were classified separately for components. In addition, drugs were classified by Biopharmaceutics Classification System (BCS) based on solubility and permeability reported in previous literature. BCS classification was defined as below (Löbenberg and Amidon, 2000):

• BCS class I: high solubility, high permeability
• BCS class II: low solubility, high permeability
• BCS class III: high solubility, low permeability
• BCS class IV: low solubility, low permeability

### Statistical Analysis

Statistical analysis of the BE results was conducted in two steps. First, post-hoc estimation of the intrasubject coefficient of variation (CV) for C_max_ and AUC_last_ was conducted for the number of subjects, and 90% confidence intervals were determined for each study. The point estimate (PE) of each PK parameter was calculated as the geometric mean of the lower limit (CI_lower_) and upper limit (CI_upper_) of the confidence interval $\left(\sqrt{{\text{CI}}_{\text{lower}}\cdot {\text{CI}}_{\text{upper}}}\right)$ considering the log-transformation recommended in BE analysis. Margin of error on a log scale was calculated as the difference of the natural logarithm of PE and the lower limit of the CI $\left[\Delta =\ln\left(\text{PE}\right)-\ln\left({\text{CI}}_{\text{lower}}\right)\right]$. Mean squared error (MSE) and intrasubject CV were calculated using the following equations (Chung et al., 2018):

$$\text{MSE}=2\cdot {\left(\frac{\Delta }{\sqrt{\frac{1}{n_{1}}+\frac{1}{n_{2}}}\cdot t_{1-2\alpha ,n_{1}+n_{2}-2}}\right)}^{2}$$

$$\text{Intrasubject}\;\text{CV}\;\left(\%\right)=100\cdot \sqrt{e^{MSE}-1}$$

(*t*: t-values of the *student* t-distribution, α: probability of type I error (assumed 0.05), $n_{1}$, $n_{2}$: sample sizes of each group).

The second step was the pooling and conversion of the estimated intrasubject CVs to the corresponding sample sizes. The intrasubject CV was pooled for drugs with a different formulation or fasting status (e.g., metformin hydrochloride, metformin hydrochloride ER, and metformin hydrochloride ER (fed) were calculated separately) using a method described previously (Chung et al., 2018). Estimation of the sample sizes was performed with the following criteria:

• Significance level (*α*): 0.05
• Power (1 − *β*): 80 and 90% (calculated separately)
• Treatment to reference ratio: 1.05 (based on the convention) (Ring et al., 2019)
• Intrasubject CV: maximum of pooled intrasubject CV of C_max_ or AUC_last_

R version 3.6.3. (R Core Team, Vienna, Austria) and statistical package “PowerTOST” were used (Labes et al., 2020) to estimate the post-hoc intrasubject CV of the C_max_ and AUC_last_ and to estimate the sample size with the intrasubject CV.

## Results

### Overall Characteristics

A total of 143 generic drugs evaluated in 588 BE studies were included in the analysis (Supplementary Table S1). Considering the formulation and fasting status, in total 171 combinations were present, of which the intrasubject CVs were calculated for both the C_max_ and AUC_last_. Therapeutic area with the most generic drugs was the Nervous system (39 drugs), followed by the Alimentary tract and metabolism (22 drugs) and the Cardiovascular system (21 drugs). Therapeutic area with the largest number of studies was the Cardiovascular system (172 studies), followed by the Nervous system (143 studies) and Alimentary tract and metabolism (92 studies). Most of the BE studies were conducted in the fasted state (565 studies in fasted status *vs.* 23 studies in fed status). Fixed-dose combination accounted for one fourth of the total studies (155 studies) for 24 generic drugs. The formulation with the greatest number of generic drugs was the immediate-release formulation (131 drugs). Other formulations included ER (12 drugs), orodispersible (4 drugs), patch (2 drugs), power (2 drugs), CR (1 drug) and sublingual formulation (1 drug). All studies were conducted with a single-dose administration. Most studies were conducted in 2 × 2 design (139 drugs, 568 studies) and the others were conducted in 2 × 2 × 4 design (7 drugs, 20 studies).

### Number of Subjects

**FIGURE 1:**
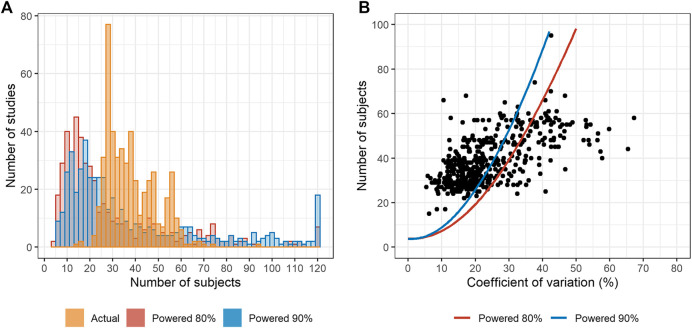
The number of subjects compared with the estimated sample size. **(A)** Distribution of the actual and number of subjects with post-hoc estimation with 80 and 90% powers. **(B)** Maximum estimated post-hoc coefficient of variation (CV) *vs.* the actual number of subjects. Dots represent the actual number of subjects. The estimated numbers of subjects are presented as solid lines. (Notes: The studies in which the estimated number of subjects was >120, the number was reduced to 120 for visualization. For fixed-dose combination, the active pharmaceutical ingredient with the highest maximum CV was selected for analysis. Only 2 × 2 bioequivalence (BE) trials were analyzed.).

The distribution of actual number of subjects differed from the estimated post-hoc sample sizes (Figure 1A). The actual number of subjects centered between 24 and 40. Most studies were conducted with more subjects than the sample sizes estimated with 90% power (Figure 1B).

**FIGURE 2:**
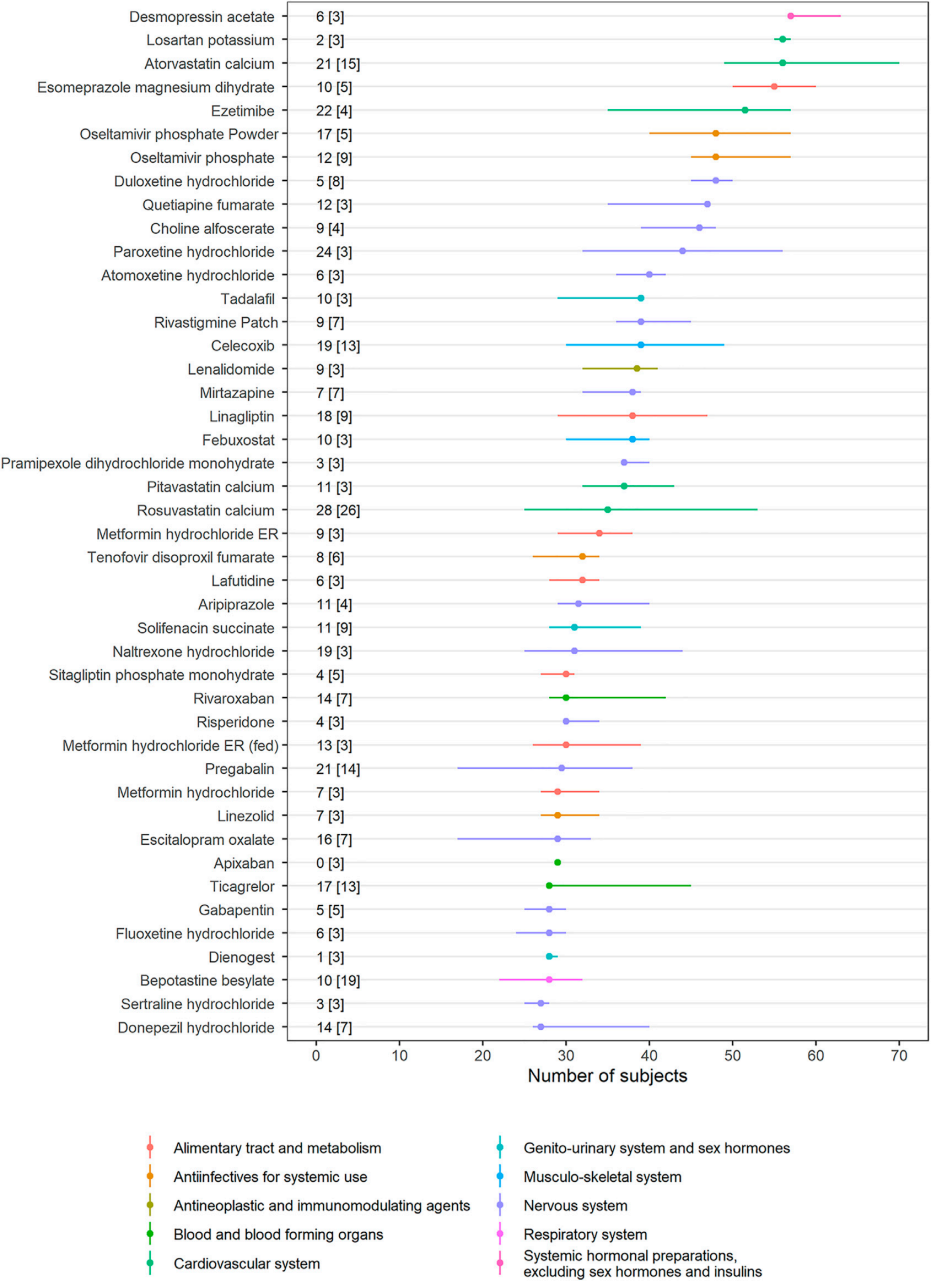
Interstudy variability of the number of subjects. Dots represent the median number of subjects in each drug, and horizontal lines represent the minimum and maximum number of subjects. The difference between the minimum and maximum number of subjects [the number of bioequivalence (BE) studies] is presented in the text. ATC classification for each drug is provided with colors (Notes: Drugs with more than three BE studies were selected. Fixed-dose combination drugs were excluded from the analysis. Only 2 × 2 BE trials are analyzed.).

For several drugs, the number of subjects was variable among studies (Figure 2). The studies on ezetimibe (*n* = 4) exhibited the highest difference of 39 subjects, whereas the studies on apixaban (*n* = 3) had the same number of subjects. Therapeutic area with the most types of drugs was the Nervous system (17 drugs), followed by Alimentary tract and metabolism (7 drugs) and the Cardiovascular system (5 drugs)*.* BE studies with 2 × 2 × 4 design were separately analyzed (Supplementary Table S2 and Supplementary Figure S1) and fixed-dose combination drugs were excluded for clarity. Intrasubject CVs for C_max_ of studies with 2 × 2 × 4 design were larger than 30% except for two studies.

### Study Duration Relative to Half-Life of the Reference Drug

**FIGURE 3:**
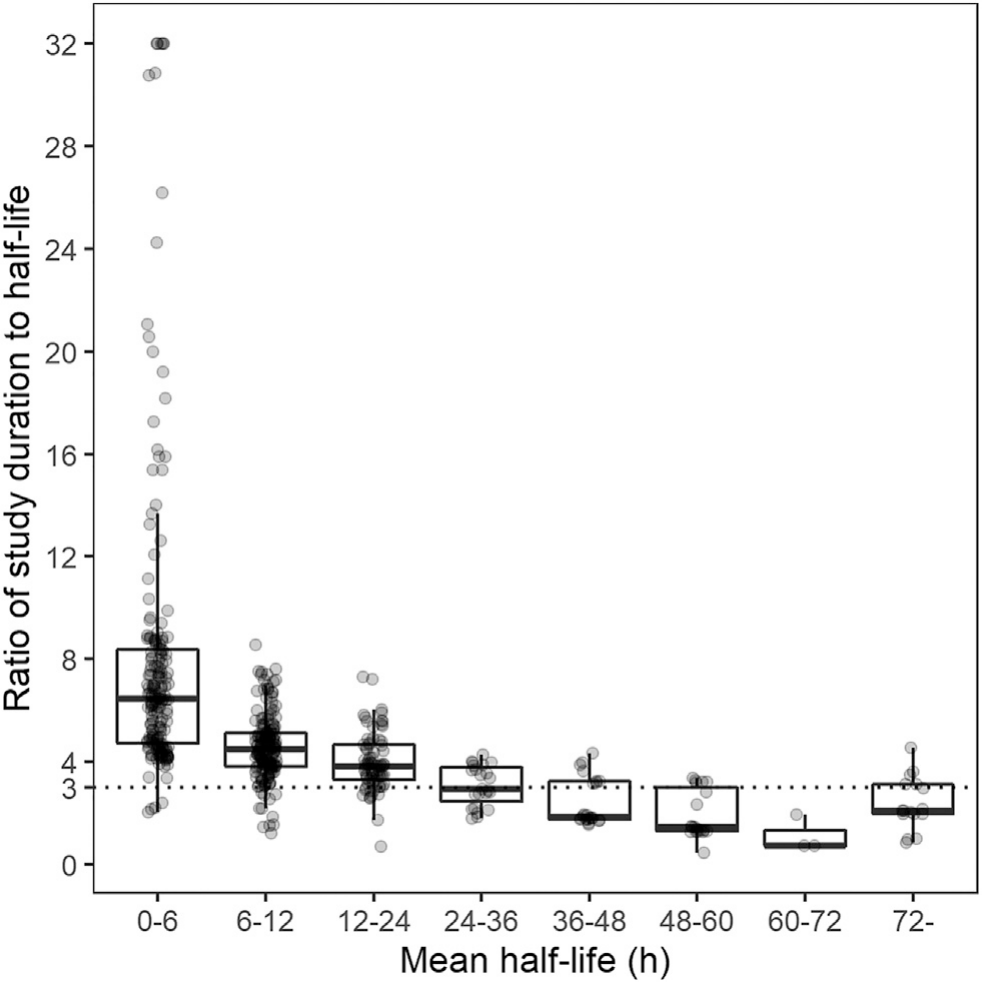
Ratio of study duration to half-life versus grouped half-life. Dotted lines represent recommended minimum ratio of study duration to half-life in South Korea. (Note: Ratios > 32 were reduced to 32 for visualization. For fixed dose combination, drug with the highest maximum coefficient of variation was selected.)

Most of the drugs exhibited terminal half-lives of less than 24 h. Study duration ranged up to 144 h and exhibited discrete distribution with an interval of 6 or 12 h. Study duration for drugs with short terminal half-lives (<24 h) was longer than three-fold of half-lives of the reference drugs. Study duration for drugs with long terminal half-lives was two- or three-fold of the half-lives of the reference drugs (Figure 3).

### Coefficient of Variation by Biopharmaceutics Classification System Classification

**FIGURE 4:**
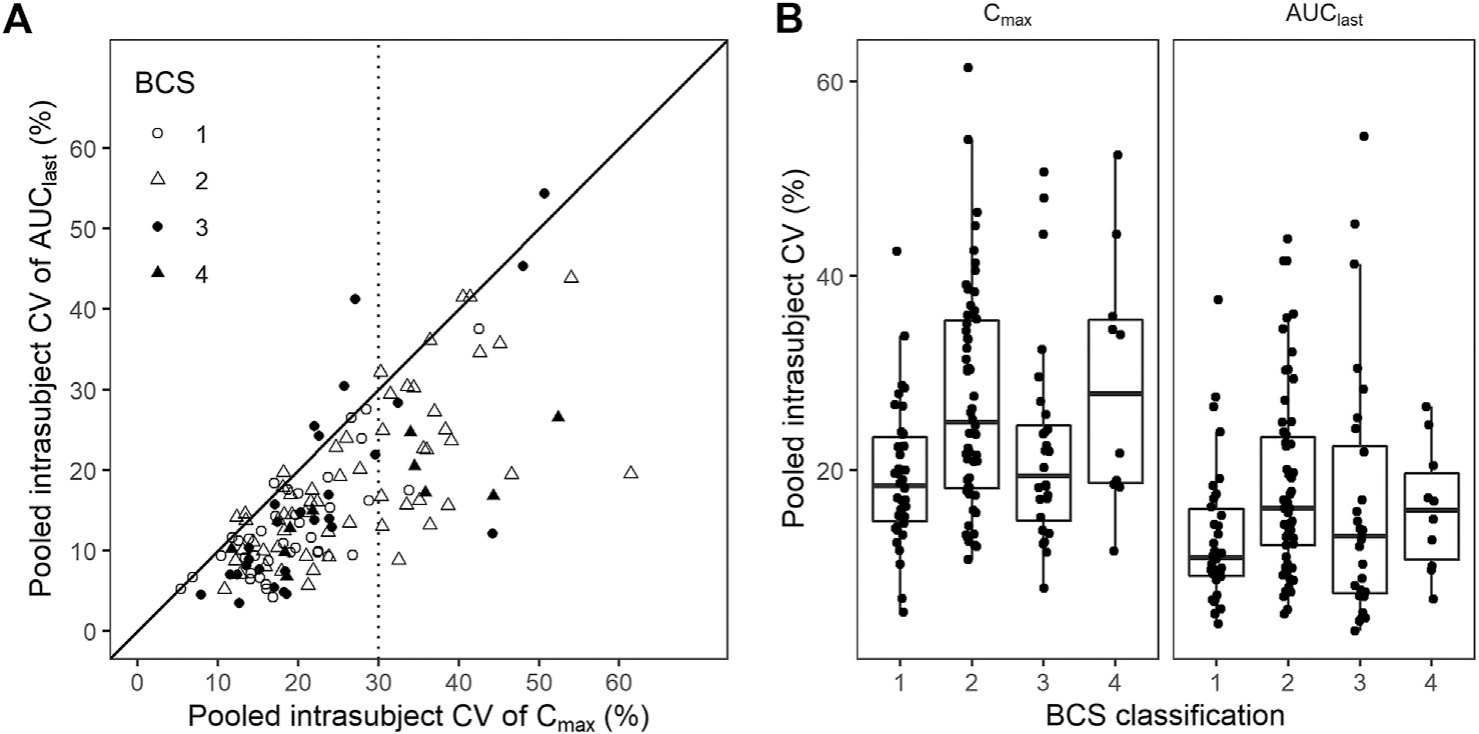
Coefficient of variation (CV) by Biopharmaceutics Classification System (BCS) classification. **(A)** Scatter plot and **(B)** box plot for pooled CV of C_max_ and AUC_last_ by BCS classification. Black solid line represents line of unity, while dotted line represents CV criterion of the highly variable drugs.

Pooled intrasubject CV calculated from C_max_ was larger than that from AUC_last_ (Figure 4A). BCS class I drugs (high solubility, high permeability) exhibited the least intrasubject CV and C_max_ exhibited a larger intrasubject CV than AUC_last_ in most cases except for oseltamivir (mean intrasubject CV for C_max_ = 37.0%), hydroxychloroquine sulfate (C_max_ = 42.6%, AUC_last_ = 37.6%), and sildenafil citrate (C_max_ = 33.3%). BCS class II and IV drugs (low solubility) accounted for most of the highly variable drugs (intrasubject CV for C_max_ > 30%) (Figure 4B).

## Discussion

In South Korea, the evaluated BE studies were performed in a similar manner. Most of the reference drugs were immediate-release oral formulations and administered in the fasted state. Therapeutic areas were focused on the Cardiovascular system, Nervous system, and Alimentary tract and metabolism, which collectively accounted for more than half of the total studies. The standard two-way crossover (2 × 2) design was adopted except for few drugs (azathioprine, carbidopa, entacapone, eperisone, naftopidil, R-thioctic acid tromethamine, and telmisartan), which adopted replicated crossover (2 × 2 × 4) design. Parallel design or partially replicated design (2 × 3 × 3) were not found.

The number of subjects mostly ranged between 24 and 40. More than half of the studies enrolled larger number of subjects than that estimated retrospectively with 90% power. In other words, the number of subjects was determined conservatively. Sample size is determined by intrasubject CV and the estimates of CV are usually referred from the previous studies. The determination of a conservative number of subjects might be attributable to higher estimated intrasubject CV. Furthermore, highly set dropout rates, which are empirically determined by the investigators, may contribute to the larger number of subjects.

We observed that conducting BE studies in South Korea is affected by specific regulatory requirements. The current BE guidelines in South Korea mandate that the minimum number of subjects in a BE study should be >12 (Ministry of Food and Drug Safety, 2018a), which was amended in September 2014 from the previous requirement of the minimum number of 12 subjects in each sequence (Ministry of Food and Drug Safety, 2014). This could affect the number of subjects for the drugs with low intrasubject CV, such as amlodipine besylate (mean CV for C_max_: 10.4%; AUC_last_: 9.4%); this drug reported the lowest number of subjects (15 subjects).

Additionally, study duration was affected by regulatory requirements. As per the current guideline, AUC_last_ should account for at least 80% of AUC extrapolated to infinity. In addition, study duration can be reduced to 72 h when a drug has a long half-life and intrasubject variability of clearance is low (Ministry of Food and Drug Safety, 2018a). In several studies, study duration was shorter than three-fold of terminal half-life. Most cases pertained to the reduced duration of 72 h, when the drugs had a long half-life. However, eight cases that did not have a drug witlong half-life had a shorter study duration. The variability of reported terminal half-life of the reference drug or effective half-life might be the possible cause (Boxenbaum and Battle, 1995).

Intrasubject CV exhibited some variability among studies. Besides the intrinsic randomness of the intrasubject CVs, different assay methods or distributions of baseline characteristics, such as demographics or genetic polymorphisms in drug metabolizing enzymes could be considered as sources of variability (Bebia et al., 2004). Several drugs exhibited intrasubject CV different than that reported in earlier studies. For example, contrary to the reported high variability of levothyroxine in a previous study (Meredith, 2003), intrasubject CV of levothyroxine was significantly low (7.1–11.5%) in our data. This might be attributable to the factors other than the randomness of the intrasubject CVs.

However, the intrasubject CV for most drugs was comparable with those reported in earlier studies. This can be supported by the fact that formulation-by-formulation effect was relatively lower than intrasubject variability (Yu et al., 2016). Furthermore, correlation between BCS class and intrasubject CV was comparable with that reported from 113 generic drugs (Sugihara et al., 2015). This comparability would support the extrapolation of intrasubject CVs reported in our study to other jurisdictions. Nonetheless, the ethnic sensitivity of BE results also needs to be investigated for extrapolation (Ozdin et al., 2020).

Our study had some limitations. The BE study reports provided by Ministry of Food and Drug Safety only included drugs that demonstrated BE, which can be a possible source of publication bias. In addition, the study reports did not include the detailed features of the study design including sampling points and safety assessments. This limited further analysis of the BE studies. Thus, further investigations on the source of interstudy variability are warranted.

## Conclusion

BE studies in South Korea were conducted in accordance with the global guideline despite the differences in details. BE studies were focused on the several therapeutic areas and conducted in similar manner. The number of subjects was generally larger than that estimated with 90% power.

## Data Availability

The datasets presented in this study can be found in online repositories. The names of the repository/repositories and accession number(s) can be found below: Ministry of Food and Drug Safety open database for approved drugs, https://nedrug.mfds.go.kr/pbp/CCBAC02/getList?totalPages=11&page=1&limit=50&sort=&sortOrder=&searchYn=&targetGb=1&title=%EC%A0%9C%EB%84%A4%EB%A6%AD.

## Supplementary Material

SUPPLEMENTARY FIGURE 1 | Interstudy variability of the number of subjects in 2 × 2 × 4 bioequivalence (BE) trials. Dots represent the median number of subjects in each drug and horizontal lines represent minimum and maximum number of subjects. Difference between the minimum and maximum number of subjects [the number of BE studies] are presented in the text. ATC classification for each drug was provided with colors. (Notes: Drugs with more than three BE studies were selected. Fixed-dose combination drugs were excluded from the analysis.)
