# Supplementary figures and images for: Current Bioequivalence Study Designs in South Korea: A Comprehensive Analysis of Bioequivalence Study Reports Between 2013 and 2019
Source: Front Pharmacol. 2021 May 4;12:651790. doi: 10.3389/fphar.2021.651790 (PMC8147690; doi:10.3389/fphar.2021.651790)

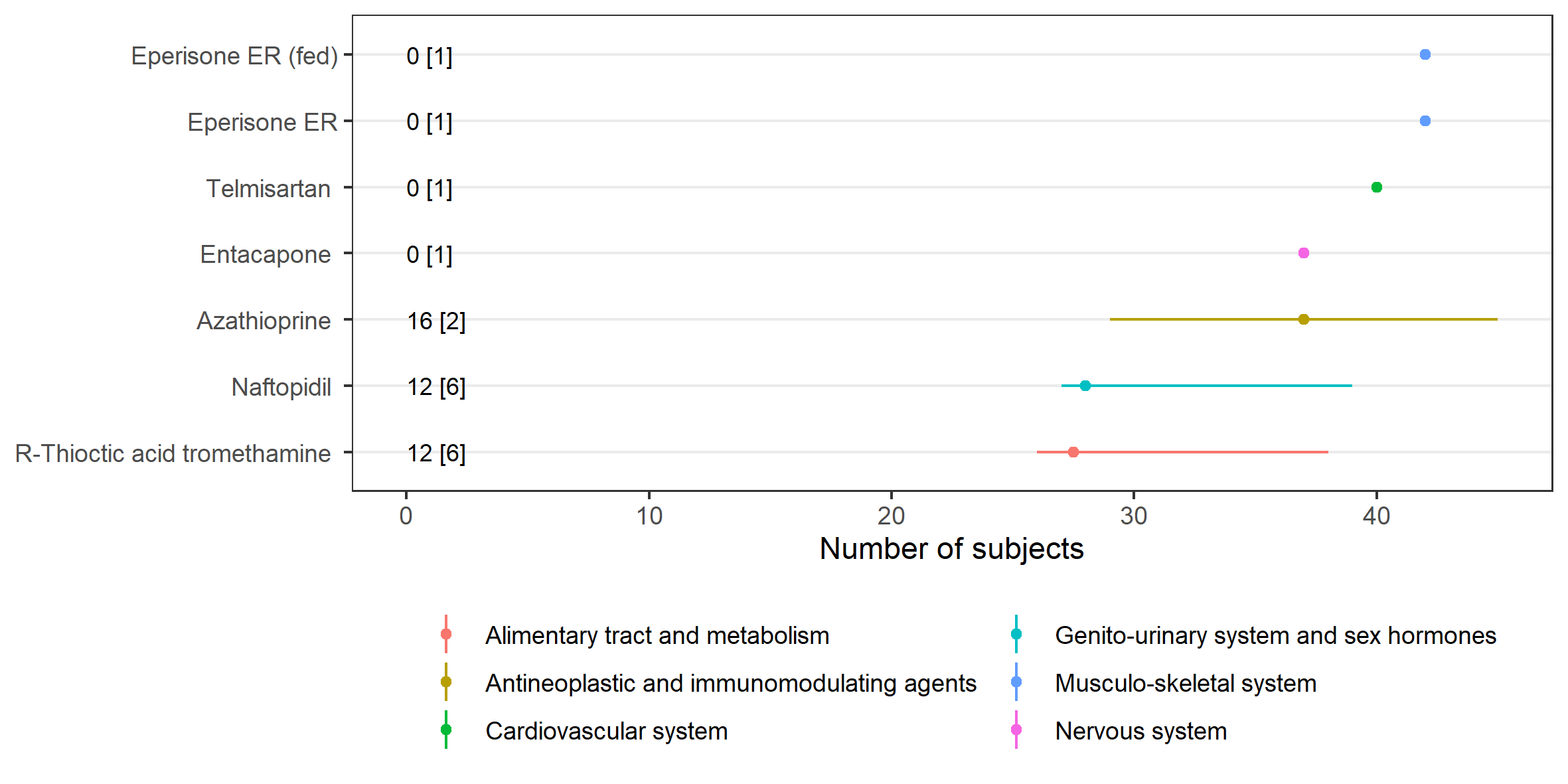

Supplement: Supplementary file 1 [file Image1.TIFF]
