# Supplementary material for: Current Bioequivalence Study Designs in South Korea: A Comprehensive Analysis of Bioequivalence Study Reports Between 2013 and 2019
Source: Front Pharmacol. 2021 May 4;12:651790. doi: 10.3389/fphar.2021.651790 (PMC8147690; doi:10.3389/fphar.2021.651790)
Supplement: Supplementary file 2 [file Table1.DOCX]

Supplementary Material

# Supplementary Table 1. Summary of the bioequivalence studies

| **WHO ATC Class**  Drug | **BCS** | **Number of studies** | **The number of subjects**^b^ | | **Mean pooled CV% of C_max_** | **Mean pooled CV% of AUC_last_** | **Required number of subjects by power** | |
| --- | --- | --- | --- | --- | --- | --- | --- | --- |
|  |  |  | **Min-max** | **Mean ± SD** |  |  | **80%** | **90%** |
| ***Alimentary tract and metabolism*** | | | | | | | | |
| Biphenyl dimethyl dicarboxylate | 2 | 2 | 37-59 | 48.0 ± 15.6 | 19.2 | 14.4 | 18 | 24 |
| Cholescalciferol Powder | 2 | 2 | 66-68 | 67.0 ± 1.4 | 14.3 | 11.1 | 12 | 14 |
| Cimetidine | 3 | 2 | 24-26 | 25.0 ± 1.4 | 13.5 | 8.2 | 10 | 14 |
| Ecabet sodium hydrate | 2 | 1 | 49 | 49 | 24.6 | 22.8 | 28 | 36 |
| Esomeprazole magnesium dihydrate | 2 | 5 | 50-60 | 54.8 ± 3.7 | 31.4 | 29.4 | 42 | 58 |
| Glimepiride | 2 | 1 | 30 | 30 | 15.6 | 10.0 | 12 | 16 |
| Itopride hydrochloride | 1 | 1 | 27 | 27 | 16.0 | 5.8 | 14 | 18 |
| Lafutidine | 2 | 3 | 28-34 | 31.3 ± 3.1 | 13.4 | 14.5 | 12 | 14 |
| Lansoprazole | 2 | 1 | 55 | 55 | 42.6 | 34.5 | 74 | 100 |
| Linagliptin | 3 | 20 | 29-47 | 40.0 ± 5.0 | 23.9 | 14.0 | 26 | 34 |
| Metformin hydrochloride | 3 | 18 | 27-46 | 36.5 ± 6.6 | 17.4 | 13.5 | 16 | 20 |
| Metformin hydrochloride ER | 3 | 3 | 29-38 | 33.7 ± 4.5 | 17.0 | 14.3 | 14 | 20 |
| Metformin hydrochloride ER (fed) | 3 | 3 | 26-39 | 31.7 ± 6.7 | 13.6 | 11.8 | 10 | 14 |
| Mosapride citrate | 2 | 1 | 33 | 33 | 18.0 | 17.8 | 16 | 22 |
| Nizatidine | 1 | 2 | 23-28 | 25.5 ± 3.5 | 20.2 | 13.4 | 20 | 26 |
| Octylonium bromide | UK | 1 | 48 | 48 | 40.6 | 56.4 | 120 | 164 |
| Pioglitazone hydrochloride | 2 | 4 | 22-36 | 29.5 ± 6.0 | 22.3 | 16.1 | 24 | 30 |
| R-Thioctic acid tromethamine | UK | 6 | 26-38 | 29.8 ± 5.0 | 49.0 | 21.0 | 94 | 128 |
| Ranitidine hydrochloride | 3 | 3 | 30-58 | 44.0 ± 14.0 | 23.7 | 16.9 | 26 | 34 |
| Rebamipide | 2 | 1 | 35 | 35 | 26.0 | 24.0 | 30 | 40 |
| S-Pantoprazole sodium trihydrate | 3 | 1 | 54 | 54 | 29.6 | 21.9 | 38 | 52 |
| Sitagliptin phosphate monohydrate | 3 | 7 | 27-31 | 28.9 ± 1.6 | 17.0 | 5.4 | 14 | 20 |
| Tripotassium bismuth dicitrate | UK | 2 | 44-58 | 51.0 ± 9.9 | 66.5 | 58.5 | 160 | 216 |
| Ursodeoxycholic acid | 2 | 2 | 37-59 | 48.0 ± 15.6 | 26.3 | 13.4 | 32 | 42 |
| ***Antiinfectives for systemic use*** | | | | | | | | |
| Cefuroxime axetil | 4 | 1 | 27 | 27 | 11.7 | 10.2 | 8 | 10 |
| Entecavir | 3 | 2 | 36-36 | 36.0 ± 0.0 | 12.4 | 7.1 | 10 | 12 |
| Fluconazole | 3 | 2 | 25-39 | 32.0 ± 9.9 | 15.2 | 7.7 | 12 | 16 |
| Irsoglandine maleate | 2 | 2 | 41-41 | 41.0 ± 0.0 | 12.1 | 8.7 | 10 | 12 |
| Itraconazole | 2 | 1 | 52 | 52 | 36.4 | 36.1 | 56 | 74 |
| Levofloxacin | 3 | 2 | 26-27 | 26.5 ± 0.7 | 18.2 | 4.8 | 16 | 22 |
| Linezolid | 1 | 3 | 27-34 | 30.0 ± 3.6 | 19.0 | 9.8 | 18 | 24 |
| Moxifloxacin hydrochloride | 1 | 2 | 38-39 | 38.5 ± 0.7 | 16.0 | 5.3 | 14 | 18 |
| Oseltamivir phosphate | 1 | 9 | 45-57 | 49.9 ± 5.1 | 37.0 | 9.4 | 58 | 78 |
| Oseltamivir phosphate Powder | 1 | 5 | 40-57 | 49.8 ± 7.3 | 23.7 | 9.3 | 26 | 34 |
| Tenofovir disoproxil fumarate | 3 | 6 | 26-34 | 31.0 ± 3.0 | 20.3 | 14.7 | 20 | 26 |
| Voriconazole | 2 | 1 | 24 | 24 | 32.5 | 8.8 | 46 | 60 |
| ***Antineoplastic and immunomodulating agents*** | | | | | | | | |
| Anagrelide hydrochloride | 4 | 1 | 47 | 47 | 21.8 | 15.0 | 22 | 30 |
| Anastrozole | 3 | 1 | 29 | 29 | 7.9 | 4.5 | 6 | 6 |
| Azathioprine | 4 | 2 | 29-45 | 37.0 ± 11.3 | 52.4 | 26.5 | 106 | 144 |
| Bicalutamide | 2 | 2 | 30-34 | 32.0 ± 2.8 | 12.3 | 14.1 | 12 | 14 |
| Erlotinib hydrochloride | 2 | 1 | 42 | 42 | 30.5 | 25.0 | 40 | 54 |
| Everolimus | 3 | 1 | 31 | 31 | 24.2 | 12.9 | 26 | 36 |
| Imatinib mesylate | 1 | 2 | 29-36 | 32.5 ± 4.9 | 18.7 | 17.6 | 18 | 22 |
| Lenalidomide | 2 | 4 | 32-41 | 37.5 ± 4.0 | 21.2 | 5.7 | 22 | 28 |
| Letrozole | 3 | 1 | 30 | 30 | 18.5 | 4.6 | 18 | 22 |
| Mycophenolate Sodium | 2 | 1 | 57 | 57 | 36.4 | 13.2 | 56 | 74 |
| Sirolimus Dispersion | 2 | 1 | 28 | 28 | 35.1 | 16.2 | 52 | 70 |
| Tacrolimus hydrate ER | 2 | 1 | 47 | 47 | 25.2 | 19.2 | 28 | 38 |
| Tacrolimus hydrate ER (fed) | 2 | 1 | 45 | 45 | 17.6 | 14.0 | 16 | 20 |
| ***Antiparasitic products, insecticides and repellents*** | | | | | | | | |
| Hydroxychloroquine sulfate | 1 | 1 | 95 | 95 | 42.6 | 37.6 | 74 | 100 |
| ***Blood and blood forming organs*** | | | | | | | | |
| Apixaban | 3 | 3 | 29-29 | 29.0 ± 0.0 | 13.8 | 8.9 | 10 | 14 |
| Camostat mesylate | 1 | 1 | 47 | 47 | 27.9 | 23.9 | 34 | 46 |
| Cilostazol | 2 | 1 | 39 | 39 | 18.2 | 19.7 | 18 | 24 |
| Clopidogrel bisulfate | 2 | 2 | 50-57 | 53.5 ± 4.9 | 45.1 | 35.7 | 82 | 110 |
| Dabigatran etexilate mesylate | 2 | 2 | 52-52 | 52.0 ± 0.0 | 40.5 | 41.5 | 70 | 96 |
| Rivaroxaban | 2 | 9 | 28-42 | 31.4 ± 4.6 | 20.9 | 14.7 | 20 | 28 |
| Ticagrelor | 4 | 13 | 28-45 | 31.0 ± 5.9 | 19.0 | 12.8 | 18 | 24 |
| ***Cardiovascular system*** | | | | | | | | |
| Amlodipine besylate | 1 | 43 | 15-61 | 44.3 ± 10.2 | 10.4 | 9.4 | 8 | 10 |
| Atorvastatin calcium | 2 | 18 | 49-70 | 56.2 ± 4.7 | 38.6 | 15.6 | 62 | 84 |
| Benidipine hydrochloride | 2 | 1 | 55 | 55 | 33.6 | 30.4 | 48 | 64 |
| Bosentan hydrate | 2 | 1 | 59 | 59 | 27.6 | 20.1 | 34 | 46 |
| Carvedilol | 2 | 1 | 46 | 46 | 33.5 | 15.7 | 48 | 64 |
| Carvedilol ER | 2 | 1 | 38 | 38 | 26.5 | 18.6 | 32 | 42 |
| Carvedilol ER (fed) | 2 | 1 | 40 | 40 | 36.6 | 20.7 | 56 | 76 |
| Choline fenofibrate | 2 | 1 | 34 | 34 | 15.9 | 8.0 | 14 | 18 |
| Choline fenofibrate (fed) | 2 | 1 | 31 | 31 | 16.1 | 8.5 | 14 | 18 |
| Dobesilate calcium | UK | 1 | 28 | 28 | 20.1 | 8.8 | 20 | 26 |
| Ezetimibe | 2 | 6 | 35-74 | 54.0 ± 13.0 | 36.0 | 22.5 | 54 | 74 |
| Hydrochlorothiazide | 4 | 6 | 25-51 | 38.5 ± 9.9 | 18.3 | 9.8 | 16 | 22 |
| Irbesartan | 2 | 2 | 39-39 | 39.0 ± 0.0 | 21.7 | 17.5 | 22 | 30 |
| Limaprost alfadex | 1 | 1 | 59 | 59 | 28.5 | 27.6 | 36 | 48 |
| Losartan potassium | 3 | 4 | 49-57 | 54.2 ± 3.6 | 44.3 | 12.1 | 80 | 106 |
| Olmesartan medoxomil | 2 | 19 | 25-41 | 35.2 ± 4.0 | 17.5 | 13.8 | 16 | 20 |
| Pentoxifylline ER | 3 | 1 | 60 | 60 | 32.4 | 28.3 | 46 | 60 |
| Pentoxifylline ER (fed) | 3 | 1 | 57 | 57 | 24.1 | 19.7 | 26 | 36 |
| Pitavastatin calcium | 1 | 3 | 32-43 | 37.3 ± 5.5 | 24.0 | 15.4 | 26 | 34 |
| Rosuvastatin calcium | 2 | 28 | 25-58 | 37.8 ± 8.6 | 21.6 | 16.1 | 22 | 28 |
| S-Amlodipine besylate dihydrate | 1 | 2 | 29-31 | 30.0 ± 1.4 | 6.8 | 6.7 | 6 | 6 |
| Simvastatin | 2 | 2 | 55-74 | 64.5 ± 13.4 | 34.3 | 30.3 | 50 | 68 |
| Telmisartan | 2 | 18 | 33-61 | 50.2 ± 7.5 | 46.5 | 19.5 | 86 | 116 |
| Trimetazidine ER | 2 | 1 | 30 | 30 | 12.7 | 7.0 | 10 | 12 |
| Trimetazidine ER (fed) | 2 | 1 | 29 | 29 | 7.7 | 5.0 | 6 | 6 |
| Valsartan | 2 | 8 | 34-58 | 47.0 ± 7.0 | 37.0 | 27.2 | 58 | 76 |
| ***Genito-urinary system and sex hormones*** | | | | | | | | |
| Dapoxetine hydrochloride | UK | 1 | 57 | 57 | 19.7 | 17.6 | 18 | 24 |
| Dienogest | 1 | 3 | 28-29 | 28.3 ± 0.6 | 14.0 | 6.5 | 12 | 14 |
| Dutasteride | 2 | 2 | 35-47 | 41.0 ± 8.5 | 17.4 | 10.4 | 16 | 20 |
| Naftopidil | 2 | 6 | 27-39 | 29.7 ± 4.6 | 61.4 | 19.6 | 140 | 188 |
| Propiverine hydrochloride | 1 | 2 | 23-28 | 25.5 ± 3.5 | 26.6 | 26.5 | 32 | 42 |
| Raloxifene hydrochloride | 2 | 1 | 54 | 54 | 38.3 | 25.0 | 62 | 82 |
| Sildenafil citrate | 1 | 2 | 45-49 | 47.0 ± 2.8 | 33.8 | 17.5 | 48 | 66 |
| Silodosin | UK | 2 | 39-48 | 43.5 ± 6.4 | 29.5 | 16.0 | 38 | 50 |
| Solifenacin succinate | 1 | 9 | 28-39 | 32.3 ± 4.1 | 11.7 | 11.6 | 8 | 10 |
| Tadalafil | 2 | 3 | 29-39 | 35.7 ± 5.8 | 13.4 | 13.7 | 10 | 14 |
| Tadalafil Orodispersible | 2 | 1 | 37 | 37 | 13.7 | 14.0 | 12 | 14 |
| Tamsulosin hydrochloride Orodispersible | 1 | 1 | 30 | 30 | 17.2 | 14.3 | 16 | 20 |
| Tamsulosin hydrochloride Orodispersible (fed) | 1 | 1 | 27 | 27 | 14.7 | 10.4 | 12 | 16 |
| Tibolone | 2 | 1 | 37 | 37 | 21.9 | 7.6 | 22 | 30 |
| Vardenafil hydrochloride | 2 | 1 | 40 | 40 | 39.0 | 23.6 | 64 | 86 |
| ***Musculo-skeletal system*** | | | | | | | | |
| Aceclofenac | 2 | 1 | 24 | 24 | 17.9 | 7.5 | 16 | 20 |
| Aceclofenac CR | 2 | 1 | 28 | 28 | 22.1 | 9.5 | 22 | 30 |
| Aceclofenac CR (fed) | 2 | 1 | 28 | 28 | 19.4 | 8.3 | 18 | 24 |
| Alendronate sodium | 3 | 1 | 55 | 55 | 50.7 | 54.3 | 114 | 154 |
| Celecoxib | 2 | 13 | 30-49 | 38.7 ± 5.3 | 30.3 | 16.7 | 40 | 54 |
| Colchicine | 3 | 1 | 47 | 47 | 22.6 | 24.3 | 28 | 36 |
| Eperisone ER | UK | 1 | 42 | 42 | 75.2 | 60.1 | 194 | 264 |
| Eperisone ER (fed) | UK | 1 | 42 | 42 | 29.5 | 21.3 | 38 | 50 |
| Febuxostat | 2 | 3 | 30-40 | 36.0 ± 5.3 | 30.4 | 13.0 | 40 | 54 |
| Loxoprofen sodium hydrate | 1 | 1 | 28 | 28 | 26.7 | 9.5 | 32 | 42 |
| Meloxicam | 2 | 2 | 29-30 | 29.5 ± 0.7 | 13.1 | 10.0 | 10 | 12 |
| Risedronate sodium | 3 | 3 | 53-68 | 62.3 ± 8.1 | 48.0 | 45.4 | 92 | 124 |
| Zaltoprofen | 2 | 1 | 29 | 29 | 23.7 | 12.3 | 26 | 34 |
| ***Nervous system*** | | | | | | | | |
| Acetaminophen | 4 | 3 | 29-35 | 33.0 ± 3.5 | 18.6 | 6.8 | 18 | 22 |
| Acetaminophen (fed) | 4 | 1 | 29 | 29 | 8.9 | 5.6 | 6 | 8 |
| Acetaminophen ER | 4 | 2 | 28-31 | 29.5 ± 2.1 | 15.3 | 5.6 | 12 | 16 |
| Acetaminophen ER (fed) | 4 | 2 | 30-32 | 31.0 ± 1.4 | 10.2 | 5.8 | 8 | 8 |
| Aripiprazole | 2 | 4 | 29-40 | 33.0 ± 4.8 | 20.9 | 9.3 | 20 | 28 |
| Atomoxetine hydrochloride | 1 | 3 | 36-42 | 39.3 ± 3.1 | 22.4 | 9.8 | 24 | 32 |
| Blonanserin | 2 | 2 | 59-61 | 60.0 ± 1.4 | 41.4 | 41.5 | 70 | 96 |
| Bupropion hydrochloride | 1 | 2 | 30-32 | 31.0 ± 1.4 | 19.6 | 10.4 | 18 | 24 |
| Buspirone hydrochloride | 2 | 1 | 46 | 46 | 54.0 | 43.8 | 112 | 152 |
| Carbidopa monohydrate | 3 | 2 | 35-37 | 36.0 ± 1.4 | 22.0 | 25.4 | 30 | 38 |
| Choline alfoscerate | 3 | 4 | 39-48 | 44.8 ± 4.3 | 27.1 | 41.2 | 70 | 94 |
| Donepezil hydrochloride | 1 | 7 | 26-40 | 30.4 ± 5.7 | 20.0 | 17.1 | 20 | 26 |
| Duloxetine hydrochloride | 2 | 8 | 45-50 | 47.9 ± 1.5 | 19.0 | 16.9 | 18 | 24 |
| Entacapone | 4 | 3 | 35-37 | 36.3 ± 1.2 | 35.8 | 17.2 | 54 | 72 |
| Escitalopram oxalate | 1 | 7 | 17-33 | 27.3 ± 5.1 | 13.8 | 11.5 | 10 | 14 |
| Fentanyl citrate Patch | 1 | 1 | 37 | 37 | 22.0 | 14.6 | 22 | 30 |
| Fentanyl citrate Sublingual | 1 | 1 | 42 | 42 | 14.6 | 9.4 | 12 | 14 |
| Fluoxetine hydrochloride | 1 | 3 | 24-30 | 27.3 ± 3.1 | 14.1 | 7.2 | 12 | 14 |
| Gabapentin | 3 | 5 | 25-30 | 28.0 ± 1.9 | 17.1 | 15.7 | 14 | 20 |
| Levetiracetam | 3 | 1 | 30 | 30 | 12.6 | 3.5 | 10 | 12 |
| Levetiracetam ER | 3 | 2 | 27-29 | 28.0 ± 1.4 | 10.4 | 8.4 | 8 | 10 |
| Levetiracetam ER (fed) | 3 | 1 | 29 | 29 | 9.1 | 4.0 | 6 | 8 |
| Levodopa | 1 | 2 | 35-37 | 36.0 ± 1.4 | 21.6 | 11.6 | 22 | 30 |
| Memantine hydrochloride | 1 | 1 | 27 | 27 | 5.4 | 5.2 | 4 | 6 |
| Methylphenidate hydrochloride | 1 | 1 | 35 | 35 | 18.1 | 10.9 | 16 | 22 |
| Mirtazapine | 1 | 7 | 32-39 | 36.0 ± 3.2 | 22.5 | 10.0 | 24 | 32 |
| Naltrexone hydrochloride | 4 | 3 | 25-44 | 33.3 ± 9.7 | 34.5 | 20.5 | 50 | 68 |
| Olanzapine Orodispersible | 2 | 1 | 26 | 26 | 10.8 | 5.2 | 8 | 10 |
| Oxcarbazepine | 4 | 2 | 45-45 | 45.0 ± 0.0 | 44.3 | 16.8 | 80 | 106 |
| Oxycodone hydrochloride ER | 1 | 1 | 44 | 44 | 12.5 | 11.2 | 10 | 12 |
| Oxycodone hydrochloride ER (fed) | 1 | 1 | 48 | 48 | 12.3 | 8.4 | 10 | 12 |
| Paliperidone ER | 2 | 1 | 54 | 54 | 30.2 | 32.1 | 44 | 60 |
| Paliperidone ER (fed) | 2 | 1 | 57 | 57 | 32.6 | 31.8 | 46 | 62 |
| Paroxetine hydrochloride | 1 | 3 | 32-56 | 44.0 ± 12.0 | 17.0 | 18.4 | 16 | 22 |
| Pramipexole dihydrochloride monohydrate | 3 | 3 | 37-40 | 38.0 ± 1.7 | 13.9 | 10.4 | 10 | 14 |
| Pramipexole dihydrochloride monohydrate ER | 3 | 1 | 37 | 37 | 19.0 | 23.8 | 26 | 34 |
| Pramipexole dihydrochloride monohydrate ER (fed) | 3 | 1 | 33 | 33 | 13.6 | 12.3 | 10 | 14 |
| Pregabalin | 1 | 14 | 17-38 | 29.7 ± 5.1 | 16.9 | 4.2 | 14 | 18 |
| Quetiapine fumarate | 2 | 3 | 35-47 | 43.0 ± 6.9 | 35.6 | 22.7 | 54 | 72 |
| Quetiapine fumarate (fed) | 2 | 1 | 39 | 39 | 42.4 | 15.8 | 74 | 98 |
| Risperidone | 2 | 3 | 30-34 | 31.3 ± 2.3 | 18.2 | 14.5 | 16 | 22 |
| Risperidone Orodispersible | 2 | 1 | 30 | 30 | 20.6 | 10.0 | 20 | 26 |
| Rivastigmine Patch | UK | 7 | 36-45 | 39.7 ± 3.3 | 15.8 | 15.3 | 14 | 16 |
| Ropinirole |  | 1 | 45 | 45 | 22.4 | 25.9 | 30 | 40 |
| Ropinirole (fed) |  | 1 | 48 | 48 | 17.3 | 16.9 | 16 | 20 |
| Sertraline hydrochloride | 1 | 3 | 25-28 | 26.7 ± 1.5 | 13.3 | 9.1 | 10 | 12 |
| Sumatriptan succinate | 3 | 1 | 30 | 30 | 22.0 | 13.8 | 22 | 30 |
| Topiramate | 3 | 3 | 27-35 | 31.7 ± 4.2 | 18.3 | 7.5 | 16 | 22 |
| Tramadol hydrochloride ER | 1 | 5 | 28-35 | 31.6 ± 3.3 | 16.3 | 8.8 | 14 | 18 |
| Tramadol hydrochloride ER (fed) | 1 | 3 | 29-32 | 30.3 ± 1.5 | 6.8 | 5.8 | 6 | 6 |
| Trazodone hydrochloride | 2 | 1 | 30 | 30 | 23.8 | 9.2 | 26 | 34 |
| Triazolam | 1 | 1 | 65 | 65 | 28.8 | 16.2 | 36 | 48 |
| Zolpidem tartrate | 1 | 1 | 26 | 26 | 23.7 | 19.1 | 26 | 34 |
| ***Respiratory system*** | | | | | | | | |
| Bepotastine besylate | 1 | 19 | 22-32 | 27.3 ± 2.4 | 15.2 | 6.7 | 12 | 16 |
| Epinastine hydrochloride | 1 | 1 | 28 | 28 | 19.7 | 14.4 | 18 | 24 |
| Montelukast sodium | 1 | 1 | 28 | 28 | 15.4 | 12.5 | 12 | 16 |
| ***Systemic hormonal preparations, excluding sex hormones and insulins*** | | | | | | | | |
| Cinacalcet hydrochloride | 4 | 1 | 47 | 47 | 34.0 | 24.7 | 50 | 66 |
| Desmopressin acetate | 3 | 3 | 57-63 | 59.0 ± 3.5 | 25.7 | 30.5 | 40 | 54 |
| Levothyroxine sodium | 3 | 2 | 38-38 | 38.0 ± 0.0 | 11.5 | 7.1 | 8 | 10 |
| ***Various*** | | | | | | | | |
| Deferasirox | 2 | 1 | 44 | 44 | 18.2 | 12.4 | 16 | 22 |

^a^**Abbreviations:** ATC, Anatomical Therapeutic Chemical Classification System; AUC_last_, area under the concentration-time curve from zero to the last measurable point; BCS, Biopharmaceutical Classification System; C_max_, maximum plasma concentration; CR, controlled-release; CV, coefficient of variation; ER, extended-release; Max, maximum; Min, minimum; SD, standard deviation; UK, unknown; WHO, World Health Organization.

^b^The actual number of subjects who were included in the pharmacokinetic analysis.
