# Supplementary material for: Current Bioequivalence Study Designs in South Korea: A Comprehensive Analysis of Bioequivalence Study Reports Between 2013 and 2019
Source: Front Pharmacol. 2021 May 4;12:651790. doi: 10.3389/fphar.2021.651790 (PMC8147690; doi:10.3389/fphar.2021.651790)
Supplement: Supplementary file 3 [file Table2.DOCX]

# Supplementary Table 2. List of the bioequivalence studies with 2 × 2 × 4 design

| **Drug** | **Study**  **number** | **Number of**  **subjects** | **Intrasubject**  **CV% of C_max_** | **Intrasubject**  **CV% of AUC_last_** | **Required number of subjects by power** | |
| --- | --- | --- | --- | --- | --- | --- |
|  |  |  |  |  | **80%** | **90%** |
| Azathioprine | #1 | 45 | 54.9% | 28.4% | 106 | 144 |
|  | #2 | 29 | 48.3% | 23.3% | 106 | 144 |
| Carbidopa monohydrate | #1 | 37 | 23.5% | 27.9% | 30 | 38 |
| Entacapone | #1 | 37 | 35.3% | 14.1% | 54 | 72 |
|  | #2 | 37 | 37.0% | 19.7% | 54 | 72 |
| Eperisone ER | #1 | 42 | 75.2% | 60.1% | 194 | 264 |
| Eperisone ER (fed) | #1 | 42 | 29.5% | 21.3% | 38 | 50 |
| Naftopidil | #1 | 28 | 61.1% | 14.8% | 140 | 188 |
|  | #2 | 28 | 63.4% | 17.9% | 140 | 188 |
|  | #3 | 27 | 51.8% | 20.3% | 140 | 188 |
|  | #4 | 39 | 69.1% | 24.3% | 140 | 188 |
|  | #5 | 28 | 59.5% | 18.3% | 140 | 188 |
|  | #6 | 28 | 59.5% | 18.3% | 140 | 188 |
| R-Thioctic acid tromethamine | #1 | 26 | 34.9% | 17.2% | 94 | 128 |
|  | #2 | 28 | 45.3% | 17.6% | 94 | 128 |
|  | #3 | 38 | 72.0% | 28.5% | 94 | 128 |
|  | #4 | 34 | 42.8% | 17.7% | 94 | 128 |
|  | #5 | 27 | 41.7% | 20.3% | 94 | 128 |
|  | #6 | 26 | 42.6% | 19.4% | 94 | 128 |
| Telmisartan | #1 | 40 | 46.5% | 23.2% | 86 | 116 |

^a^**Abbreviations:** CV, coefficient of variation.
